# Changes in Positive and Negative Affect during Acute Psychiatric Treatment in People with Social Anxiety Disorder

**DOI:** 10.1155/2023/7614830

**Published:** 2023-11-17

**Authors:** Emily M. Bowers, Andrew D. Peckham, Fallon R. Goodman, Melanie A. Hom, Erin Beckham, Thröstur Björgvinsson, Courtney Beard

**Affiliations:** ^1^Utah State University, Logan, UT, USA; ^2^McLean Hospital/Harvard Medical School, Department of Psychiatry, Belmont, MA, USA; ^3^University of South Florida, Department of Psychology, Tampa, FL, USA; ^4^Stanford University School of Medicine, Department of Psychiatry and Behavioral Sciences, Stanford, CA, USA

## Abstract

People with social anxiety disorder (SAD) experience less positive affect (PA) and more negative affect (NA) than the general population, a pattern more similar to depression than other anxiety disorders. There is a dearth of research assessing whether PA is targeted effectively during treatment, even though this is an important emotional aspect for quality of life. The primary aim of this study was to examine daily changes in self-report PA and NA among a sample of partial hospital program (PHP) patients with SAD. A secondary aim was to examine baseline depression severity as a moderator of daily PA and NA change. Patients were adults (*N* = 241) diagnosed with SAD seeking treatment at a typically 1–2-week transdiagnostic behavioral health PHP from September 2017 to September 2019. Patients completed (1) the International Positive and Negative Affect Schedule-Short Form (IPANAS-SF) each treatment day to assess affect and (2) the Patient Health Questionnaire-9 Item Version (PHQ-9) at baseline to assess depressive symptoms. Data from the first nine days of treatment were included in analyses: two-level multilevel model (MLM) analyses were used to address study aim 1, and baseline depression severity was added as a moderator to address study aim 2. Patients reported significant decreases in NA but no significant changes in PA over the course of PHP treatment. Additionally, there was no significant evidence of depression moderating NA change; however, depression did moderate PA change. These findings suggest that patients with SAD report significant decreases in NA but no change in PA over transdiagnostic group treatment, with increases in PA being stronger among patients with more severe depressive symptoms. PA captures an important emotional experience for living a fulfilling life, yet CBT-based treatments may not be effectively improving these areas. Future studies targeting PA in SAD treatment can inform whether doing so ultimately improves treatment outcomes for SAD.

## 1. Introduction

Social anxiety disorder (SAD) is characterized by a marked fear of social situations and judgment from others. SAD is the fourth most common psychiatric illness among adults in the United States, with an estimated 12.1% lifetime prevalence [1–3], and one of the most common worldwide [4]. Social anxiety can lead to significant impairment: people with SAD are more likely to experience poorer quality of social relationships [5, 6], lower quality of life [7], and increased suicidal ideation and behavior [8, 9] compared to those without SAD. Furthermore, SAD can lead to substantial financial burden and occupational impairment [10]. For instance, people with more severe social anxiety symptoms report lower workplace productivity, leading to increased economic costs [11]. The extensive impact of SAD highlights the importance of effectively treating this debilitating disorder.

Treatments for SAD—particularly cognitive behavioral therapy (CBT)—are relatively effective [12, 13]. Yet, people with SAD are less likely to experience full remission of symptoms than individuals with other anxiety disorders or major depression, independent of treatment received [14]. Further research is needed to understand how CBT reduces social anxiety symptoms and to identify novel treatment targets among people with SAD.

### 1.1. Social Anxiety and Affect

Positive affect (PA) and negative affect (NA) are related but distinct constructs; they are independent emotional experiences rather than opposite ends of a single continuum of emotion [15, 16]. Indeed, whereas elevated NA is a core feature of emotional disorders [17, 18], chronically low positive affect (PA) is not consistently associated with emotional disorders. SAD is distinct from other anxiety disorders in that it is characterized by both high NA and low PA [19], demonstrating an affective profile similar to depression. In a meta-analysis of 19 studies (*n* = 2,976), social anxiety symptom severity and PA were moderately negatively correlated (*r* = −.21; 95% CI: -.16 to -.26) [20], and this association remains even after controlling for comorbid depression symptoms.

The affective profile of SAD provides a useful intervention target in psychotherapy. Current psychological treatments for social anxiety lead to decreases in NA in people with SAD, but findings on changes in PA are mixed [21, 22]. In Sewart and colleagues' study comparing the effectiveness of individually delivered CBT and acceptance and commitment therapy (ACT) for SAD, participants in both treatment conditions experienced significant decreases in NA and significant increases in PA from pre- to post-treatment. However, the observed PA increases in the treatment groups did not significantly differ from those observed among those in a waitlist condition. In a pilot study examining the effects of an 8-week group Mindfulness-Based Cognitive Therapy (MBCT) intervention on PA and social anxiety symptoms among 22 adults with SAD, PA significantly increased from pre- to posttreatment [22]. Moreover, these increases in PA, but not decreases in NA, predicted social anxiety improvement [22]. One explanation for these findings is that increases in PA during treatment might increase social approach behavior and facilitate social benefits [23]. Additionally, targeting PA through acts of kindness also led to social benefits as social approach increased and social avoidance decreased [23]. Beyond these studies, PA changes in SAD treatment have been relatively understudied.

### 1.2. Social Anxiety and Depression Comorbidity

As noted, the profile of high NA and low PA is also present in depression, suggesting that there may be shared mechanisms underlying both depression and SAD [18, 19, 24, 25]. Social anxiety precedes a depressive episode in about 70% of the comorbid cases [3]. The high rates of comorbidity between SAD and depression are important when assessing treatment outcomes, as comorbid SAD and depression predict greater symptom severity and poorer prognosis [3, 26, 27]. Indeed, the mechanisms involved in the development and maintenance of both SAD and depression have been a recent area of research focus [28–31]. A meta-analysis found a significant association between more severe baseline depressive symptoms and a greater reduction in social anxiety symptoms from pre- to post-treatment [32]; these findings suggest that greater reductions in depression may be related to decreases in social anxiety. Given that SAD and depression may share similar mechanisms that maintain and exacerbate symptoms, it is important to examine how depression may impact treatment outcomes in people with social anxiety.

### 1.3. The Present Study

Although SAD has been associated with low PA and high NA, very few studies have examined changes in PA and NA during treatment. The primary aim of this study was to examine daily changes in self-reported PA and NA among an acute sample of CBT-based partial hospital program (PHP) patients with a current SAD diagnosis. We hypothesized that self-reported NA would significantly decrease and self-reported PA would increase during PHP treatment, consistent with prior literature. A secondary aim of the study was to examine self-reported depression symptoms as a moderator of NA and PA change in patients with SAD, given literature suggesting that comorbid depression and social anxiety may impact treatment outcomes. Due to the exploratory nature of these aims, we did not posit any *a priori* hypotheses. Of note, this study is an analysis of a larger clinical dataset of patients receiving treatment at the PHP; symptom measures are completed as part of standard clinical care, and data collection has been ongoing for the past decade (see [33, 34] for detailed descriptions of PHP treatment and data collection).

## 2. Methods

### 2.1. Participants and Treatment Setting

Participants were adult (age 18+) patients seeking acute psychiatric treatment at a transdiagnostic PHP in the Northeastern United States from September 2017 to September 2019. PHPs provide a unique opportunity to examine short-term affective change during acute psychiatric treatment; patients participate in treatment for six hours daily Monday through Friday for approximately two weeks, although length of treatment varies depending on the patients' clinical response and functioning. Given that SAD often cooccurs with other disorders (e.g., depression, eating disorders, and psychosis) [35–37], the transdiagnostic treatment setting represents an ideal context in which to examine the present study aims. This PHP is housed within a nonprofit, freestanding psychiatric hospital. PHP treatment focused on stabilization of symptoms via CBT-, dialectical behavior therapy- (DBT), and ACT-based group therapy, along with pharmacological treatment. See Forgeard et al. for an overview of the PHP, and see Beard et al. for a thorough description of eligibility for the PHP [34, 38].

PHP patients were eligible for this study if they provided written informed consent to participate in research and met criteria for a current diagnosis of SAD at PHP admission (see Section 2.2). Comorbid diagnoses were not an exclusion criterion.

### 2.2. Measures

#### 2.2.1. Mini-International Neuropsychiatric Interview (MINI) Version 7.0.2

The MINI is a structured diagnostic assessment used to assess psychiatric diagnoses [39]. During their first therapy session at the PHP, patients were assessed for major depressive episode, manic and hypomanic episodes, panic disorder, agoraphobia, social anxiety disorder, obsessive compulsive disorder, substance use disorders, psychotic disorders and mood disorders with psychotic features, bulimia nervosa, binge eating disorder, and generalized anxiety disorder. The MINI demonstrates good psychometric properties, including excellent interrater reliability for mood and anxiety disorders [39]. The MINI was administered to participants by trained staff psychologists, postdoctoral fellows, and graduate-level interviewers. MINI training included review of administration manuals with postdoctoral fellows, mock interviews, and monthly supervision that included reliability rating of recorded interviews.

#### 2.2.2. International Positive and Negative Affect Schedule-Short Form (IPANAS-SF)

The IPANAS-SF is a brief, 10-item self-report measure assessing PA and NA frequency [16]; items represent a subset of the full PANAS [40]. Patients were asked to rate their affect (upset, hostile, alert, ashamed, inspired, nervous, determined, attentive, afraid, and active) over the past 24 hours each morning during PHP treatment on a 5-point Likert scale from 1 (“Never”) to 5 (“Always”). NA and PA were scored separately; items are averaged to yield total scores ranging from 1 to 5, with higher scores indicating greater NA or PA, respectively. The IPANAS-SF has demonstrated acceptable reliability and validity [16]. The IPANAS-SF NA (*α*s = .74 to .89) and PA (*α*s = .76 and .83) subscales demonstrated acceptable to good internal consistency across time points in this sample.

#### 2.2.3. Patient Health Questionnaire-9 Item Version (PHQ-9)

Patients completed the PHQ-9 at admission^1^. The PHQ-9 is a 9-item self-report questionnaire assessing depression symptom severity [41]. Patients rate how frequently they experienced each symptom in the prior two weeks (e.g., “Feeling tired or having little energy?”) using a 4-point Likert scale ranging from 0 (“Not at all”) to 3 (“Nearly every day”). Total scores range from 0 to 27; higher scores indicate greater depression symptom severity. Within our PHP setting, the PHQ-9 has been identified as a valid depression severity measure [42] and demonstrated acceptable internal consistency at PHP admission (*α* = 0.88).

### 2.3. Procedures

All study procedures were approved by the hospital's Institutional Review Board. Patients completed the above measures as part of routine clinical care while attending the PHP. Self-report measures were administered via Research Electronic Data Capture (REDCap), a secure, web-based application designed to capture and store research data [43].

### 2.4. Data Analytic Approach

To address study aims, analyses were conducted in R [44] with the lme4 package [45] by the second author (A.D.P.). Only patients who met criteria for current SAD on the MINI were included. Due to the variability of treatment days in the PHP and significant missing self-report data after the ninth day of treatment, we included the first nine days of treatment in our multilevel model (MLM) affect analyses to have a comparable amount of data for each person (consistent with other studies conducted in this setting; for example, [46]). Two-level MLM analyses were used to test daily affect change within each individual over the first nine days of PHP treatment, with separate models for NA and PA. For MLM analyses, parameters of linear growth models were compared to a no-growth model, and models were fit using restricted maximum likelihood (REML). REML was also chosen to accommodate the fact that these models are suitable for accommodating missing data (i.e., daily negative and positive affect values). Data were assumed to be missing at random, and missing data ranged from 0% to 6.22% for treatment days 1-6 and 12.9% to 39.0% for treatment days 7-9. Traditional model fit indices (e.g., AIC) were used to evaluate model fit. For aim 2, baseline depression severity (mean-centered PHQ-9 scores obtained at admission) were added as a moderator to each MLM growth model.

## 3. Results

Participants in the final sample included 241 adult patients^2^ (*M*_age_ = 28.72 ± 11.21 years) that identified as predominantly female (*n* = 131, 54.4%) and non-Hispanic White (*n* = 221, 91.7%); see Table 1 for detailed demographics. Notably, 167 patients (69.3% of the sample) were diagnosed with current major depressive episode in addition to social anxiety disorder; see Table 2 for other baseline clinical characteristics.

### 3.1. Aim 1: Daily Affect Change over Treatment

#### 3.1.1. Negative Affect

Compared to an initial no-growth model (AIC: 4407.82, BIC: 4425.19), the linear growth model for daily NA showed better fit indices (AIC: 4036.08, BIC: 4070.83). Results of the linear growth model indicated that the mean of the intercept (2.83, SE: .04, 95% CI: 2.74–2.91) was significant (*t*(2121) = 64.89, *p* < .001). In addition, the slope was significant (-0.073, SE: .005, 95% CI: -.083 – -.062), *t*(2121) = −13.55, *p* < .001), showing a decrease in negative affect over the course of PHP treatment.

#### 3.1.2. Positive Affect

Compared to a no-growth model (AIC: 3767.09, BIC: 3784.46), the linear growth model for daily PA showed modestly better fit indices (AIC: 3695.59, BIC: 3730.33). The mean intercept for this model (2.31, SE: .04, 95% CI: 2.23–2.38) was significant (*t*(2117) = 59.57, *p* < .001); however, the slope was not (.005, SE: .005, 95% CI -.005–.015, *t*(2117) = 1.06, *p* = .29).

### 3.2. Aim 2: Depression as a Moderator of Affect Change

**Figure 1:**
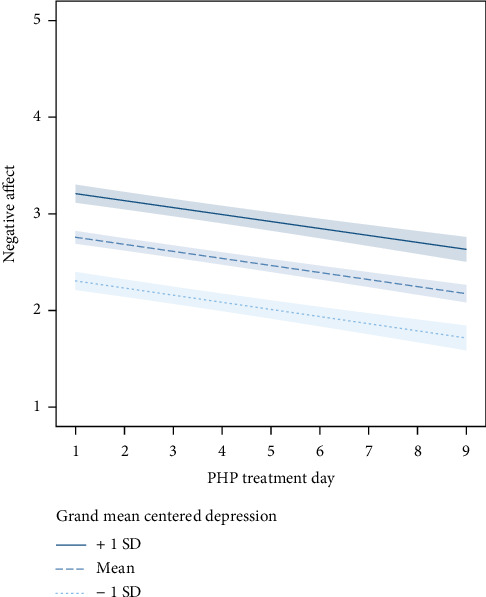
Daily changes in negative affect during treatment with no moderation by baseline depression.

We analyzed NA and PA changes using baseline PHQ-9 depression symptom severity as a moderator. The addition of depression severity improved model fit for the NA growth model (AIC = 3871.42, BIC = 3917.66), as well as the PA growth model (AIC = 3645.7, BIC = 3691.93). Depression severity was positively and significantly associated with level of NA (unstandardized Beta = .08 (SE = .01), *t*(296) = 12.6, *p* < .001); however, there was no evidence of an interaction between depression severity and treatment day (Beta = .0002 [SE = .001], *t*(2097) = .16, *p* = .83), suggesting that initial depression severity had no significant effect on change in NA over time. Results of this model are shown in Figure 1.

**Figure 2:**
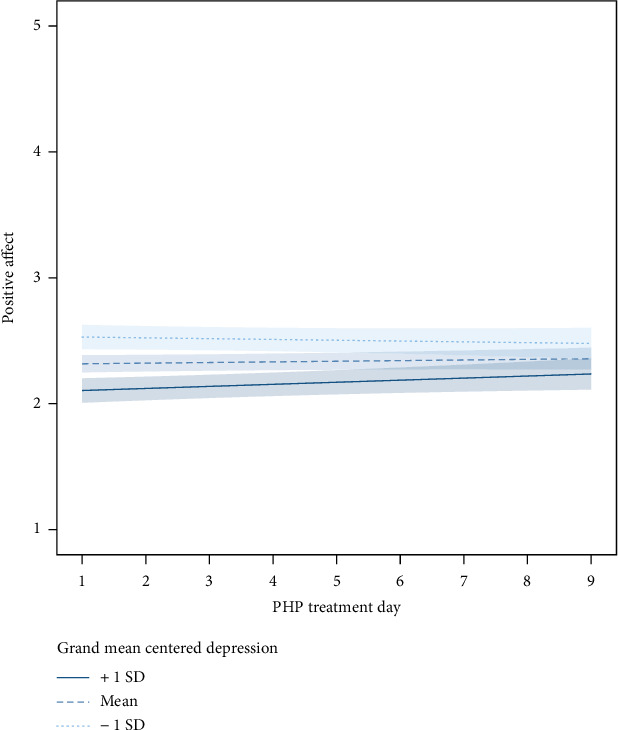
Daily changes in positive affect during treatment moderated by baseline depression.

In the PA model, depression severity was negatively and significantly associated with level of PA (Beta = −.04 [SE = .007], *t*(296) = −6.06, *p* < .001). In addition, there was a significant interaction of depression severity and treatment day (Beta = .002 [SE = .0009], *t*(2094) = 2.24, *p* = .025), indicating that higher initial depression severity was associated with a greater increase in PA over time. Results of this model are shown in Figure 2.

## 4. Discussion

This study aimed to examine NA and PA changes over the course of PHP treatment among acute psychiatric patients with SAD. Findings revealed that patients reported a significant linear decrease in daily NA throughout treatment but did not report significant changes in PA. Additionally, although depression severity at PHP admission did not moderate the trajectory of NA during treatment, it did significantly moderate the trajectory of PA, such that more severe depression symptoms were associated with a greater increase in PA over time.

As hypothesized, patients with SAD reported significant decreases in NA over the course of treatment. This result is not surprising given that most traditional psychotherapies for depression and anxiety disorders focus on reducing NA (e.g., anxiety) and consistently demonstrate effectiveness in doing so [21, 47]. In contrast, we found that low PA remained relatively stable, on average, among our samples throughout treatment. This pattern of findings aligns with the conceptualization of NA and PA as distinct emotional systems and adds to a growing literature revealing that PA may not change for patients with SAD during treatment [47–49]. Findings on PA changes have been equivocal, however. One explanation for these mixed results is that treatments typically focus on decreasing NA, specifically; yet, when PA is a primary treatment target, PA levels increase [47, 49]. For example, in a study of 142 socially anxious individuals, Alden and Trew found significant increases in PA among those who were asked to engage in acts of kindness towards others, compared to those in an experimental condition targeting social safety behaviors and those in a control tracking condition [23]. Incorporating social approach behaviors in CBT for social anxiety may enhance relational functioning, thus facilitating increases in PA [50]. In a broader sample of participants with depressive and anxiety symptoms, positive affect treatment (PAT) has demonstrated promising results in both increasing PA (*p* = .009, *d* = .52) and decreasing NA (*p* = 0.33, *d* = .52) compared to negative affect treatment [47] by targeting reward system deficits. Although PAT has not been evaluated in a social anxiety sample, this research provides evidence on the utility of targeting reward activities (e.g., positive event scheduling, savoring, gratitude) in clinical populations. Together, this research suggests that incorporation of PA-specific activities may be useful to explore in SAD treatment to address PA deficits.

Another possible explanation for our pattern of unchanged PA results could be the method PA was assessed in our study. Specifically, all positive emotions were from the high-arousal domain (i.e., alert, inspired, determined, attentive, and active). Low-arousal indicators of PA may be more salient among individuals with SAD. For instance, a study by Cohen and Huppert compared global PA and specific positive emotions (e.g., joy, pride, love, and awe), and they found that experiencing pride was significantly negatively associated with social anxiety [51]. It is also possible that PA may change more slowly than NA and gradually increase over time following treatment, a change that we could not detect over the course of nine days. To our knowledge, studies have yet to evaluate rates of change of PA among individuals with social anxiety. Moving forward, research is needed to better understand what positive emotions are salient in SAD treatment and if certain types of PA are effectively targeted by conventional SAD treatments (e.g., CBT and ACT).

Finally, it was notable that depression moderated PA change in our sample. Specifically, SAD patients with higher initial depression symptom severity showed greater changes in PA over the course of treatment. Given that depression severity is negatively associated with PA (i.e., those with more severe depression demonstrate lower PA), this result might, in part, reflect that those with more severe depression symptoms had greater potential for improvements in PA. Interestingly, however, baseline depression symptom severity did not moderate NA changes over the course of treatment among our patients with SAD. Since SAD often precedes depressive episodes [3], perhaps the social interaction inherent in PHP participation (e.g., attending up to five groups a day, frequent meetings with treatment providers, and connection in a milieu space) effectively targeted social anxiety and related negative affect via exposure. These interactions may have driven the decreases in NA regardless of baseline depression. Additional research is needed to better understand the mechanisms underlying these moderation results.

### 4.1. Limitations and Future Directions

The present study had several limitations that provide opportunities for future research. First, although we were examining patients with SAD, we did not include any direct measures of social anxiety at discharge to assess SAD symptom improvement. Second, though the acute population was a strength and offered a clinically heterogeneous sample, we were unable to compare our naturalistic PHP sample to a control condition. Future studies could incorporate a control or waitlist condition to address this limitation. Third, people with SAD often do not seek treatment [52, 53], so our results might not generalize to individuals with SAD at large. Further, our sample included participants who consented to participate in research; therefore, our findings may not be generalizable to all people with SAD accessing treatment. Fourth, the present study relied on self-report measures assessing symptoms over the past two weeks (depression) or past 24 hours (affect); self-report responses may be susceptible to recall biases in people with SAD [54]. Future studies could incorporate clinical evaluators who are unaware of study aims to measure multiple modalities of symptom change. Further, the self-report affect measures had substantial missing data as the treatment progressed (missing values range from 12.9% on day 7 to 39.0% on day 9). Lastly, our sample had limited ethnic, racial, and cultural diversity. It is essential to examine affect change in a more diverse social anxiety sample to better inform future treatment.

## 5. Conclusion

In conclusion, our results demonstrate that patients with SAD experience decreases in NA, but no change in PA during acute psychiatric treatment at a PHP. Our results also suggest that depression may moderate PA change, but not NA change over the course of treatment. Such findings suggest that CBT-based approaches can be enhanced to further address low PA in people with social anxiety. PA (e.g., feeling alert, inspired, determined, attentive, and/or active) captures an important emotional experience for living a fulfilling life, yet we may be failing people with SAD by not effectively improving these areas with CBT-based treatments for social anxiety. It is important to evaluate PA in future treatment progress tracking as well as in clinical research studies to more adequately address this need and potentially enhance patients' quality of life as treatment for social anxiety develops. It may be useful to emphasize increasing PA alongside decreasing NA, although research is needed to understand why and how PA can change in people with SAD. Future studies targeting PA in SAD treatment can inform whether doing so ultimately improves treatment outcomes for SAD.

## Figures and Tables

**Table 1:** Participant sociodemographic characteristics.

	Full sample (*N* = 241)	
	*n*	Valid (%)
Gender identity		
Female	131	54.4%
Male	107	44.4%
Nonbinary	2	0.8%
Genderqueer	1	0.4%
Sexual orientation		
Bisexual	45	18.7%
Gay/lesbian	15	6.2%
Heterosexual/straight	162	67.2%
Queer	9	3.7%
Asexual	3	1.2%
Pansexual	3	1.2%
Questioning	3	1.2%
Missing	1	0.4%
Age	*M* = 28.7 SD = 11.2 Range = 18‐71	
Ethnicity		
Hispanic or Latin/o/a/x	14	5.8%
Race^1^		
American Indian/Alaskan Native	3	1.2%
Asian	7	2.9%
Black	6	2.5%
Native Hawaiian/Pacific Islander	0	0.0%
White	221	91.7%
Jewish	1	0.4%
Latin American	1	0.4%
Mexican	1	0.4%
Do not know	6	2.5%
Education level		
Some high school or less	1	0.4%
High school/GED	24	10.0%
Some college/associate degree/trade school	106	44.0%
Four-year college	68	28.2%
Postcollege	42	17.4%
Marital status		
Never married	176	73.0%
Separated/divorced	10	4.2%
Widowed	2	0.8%
Married	45	18.7%
Living with partner	8	3.3%

^1^Categories not mutually exclusive.

**Table 2:** Descriptive statistics for self-report measures.

	Pretreatment (T1)					Posttreatment (T2)				
	*M*	SD	Min	Max	*α*	*M*	SD	Min	Max	*α*
PHQ-9 depression symptoms	15.62	5.49	2	26	.88^1^, .85^2^	10.54	5.11	1	25	.86^1^, .84^2^
IPANAS-SF positive affect	2.33	.77	1	4.80	.77	2.68	.93	1	5.0	.90
IPANAS-SF negative affect	2.99	.78	1	4.60	.74	2.23	.83	1	4.8	.78

^1^From the standard PHQ-9. ^2^From the disaggregated version of the PHQ-9. *Note.* PHQ-9 = Patient Health Questionnaire-9 Item Version; IPANAS-SF = International Positive and Negative Affect Schedule-Short Form.

## Data Availability

The naturalistic data used to support the findings of this study have not been made available due to sensitive clinical information and hospital policy.

## Endnotes

^1^For a subgroup of participants (*n* = 75), the PHQ-9 version administered was disaggregated such that several items were split into subcomponents (e.g., sleep item was split into “Trouble staying asleep?” and “Trouble falling asleep?”). Disaggregated PHQ-9 scores were then re-aggregated for analysis in this study and demonstrated acceptable internal consistency (*α* = 0.85). ^2^During the study time frame, 92% of patients attending the PHP consented to participate in research. No significant differences (*p*s > .05) were found on gender, age, sexual orientation, and Hispanic ethnicity between those who consented (*n* = 241) and those who did not (*n* = 22); however, significantly (*p* < .01) more white participants are present in the consented sample.
